# The Elements of Materia Medica and Therapeutics

**Published:** 1853

**Authors:** 


Art. XIII.—*The Elements of Materia Medica and Therapeutics.* By Jonathan Pereira, M. D., F. R. S. and L. S. Third American Edition, enlarged and improved by the Author. Including notices of most of the Medicinal Subtances in use in the civilized world, and forming an Encyclopedia of Materia Medica. Edited by Joseph Carson, M. D., Professor of Materia Medica and Pharmacy in the University of Pennsylvania, Fellow of the College of Physicians of Philadelphia, etc. Vol II. Philadelphia: Blanchard & Lea. 1854. Pp. 1226, 8vo.

All that we propose to do, it simply to announce the publication of the second volume of this great work. An analysis of its rich pages is out of the question, and would be a work of supererogation if it were practicable within any space at our command; this treatise is an encyclopedia, and must be consulted by each reader for himself. It is a full and systematic summary of all that the practitioner or student of medicine can wish to know of the materia medica. It is melancholy to think that the author of this great monument of learning and patient industry has not been spared to reap the rewards of his honorable toil. Few books have been published in our time so comprehensible and full, and evincing accurate knowledge in regard to so vast a variety of subjects.

